# Linking social cognition with social interaction: Non-verbal expressivity, social competence and "mentalising" in patients with schizophrenia spectrum disorders

**DOI:** 10.1186/1744-9081-5-6

**Published:** 2009-01-23

**Authors:** Martin Brüne, Mona Abdel-Hamid, Claudia Sonntag, Caroline Lehmkämper, Robyn Langdon

**Affiliations:** 1 Department of Psychiatry, LWL-University Hospital, University of Bochum, Bochum, Germany; 2 Macquarie Centre for Cognitive Science, Macquarie University, Sydney, Australia

## Abstract

**Background:**

Research has shown that patients with schizophrenia spectrum disorders (SSD) can be distinguished from controls on the basis of their non-verbal expression. For example, patients with SSD use facial expressions less than normals to invite and sustain social interaction. Here, we sought to examine whether non-verbal expressivity in patients corresponds with their impoverished social competence and neurocognition.

**Method:**

Fifty patients with SSD were videotaped during interviews. Non-verbal expressivity was evaluated using the Ethological Coding System for Interviews (ECSI). Social competence was measured using the Social Behaviour Scale and psychopathology was rated using the Positive and Negative Symptom Scale. Neurocognitive variables included measures of IQ, executive functioning, and two mentalising tasks, which tapped into the ability to appreciate mental states of story characters.

**Results:**

Non-verbal expressivity was reduced in patients relative to controls. Lack of "prosocial" nonverbal signals was associated with poor social competence and, partially, with impaired understanding of others' minds, but not with non-social cognition or medication.

**Conclusion:**

This is the first study to link deficits in non-verbal expressivity to levels of social skills and awareness of others' thoughts and intentions in patients with SSD.

## Background

Over the last 25 or so years, research into the nonverbal behaviour of patients with schizophrenia or schizophrenia spectrum disorders (SSD) has demonstrated that patients can be reliably distinguished from unaffected individuals on the basis of their reduced expressivity [1-3]. For example, microanalytic studies of facial movements using the Facial Action Coding System (FACS) [4] revealed that patients with schizophrenia are reduced in their facial expressivity, particularly with regards to the expression of positive emotions that are usually encoded via movements of the upper part of the face [2,5]. Other studies carried out in more "naturalistic" settings on hospital wards have shown that persons with schizophrenia avoid physical proximity to others and display other deficits in engaging in social interaction [1,6,7]. These studies have in common that they are based on ethological methodology. Ethological studies in normal populations have shown that humans use facial expressions, gestures and whole body movements to convey communicative signals that invite social interaction (affiliation), reduce aggression through appeasement (submission), imply motivational ambivalence (fight or flight) or escalate social competition [8-10]. These nonverbal signals during social interaction are human universals that are to a great deal encoded and understood in similar ways across cultures.

Since ethological studies have proven difficult to pursue in psychiatric populations because they are fraught with time-consuming analyses, a simpler method to examine videotaped nonverbal behaviour in clinical settings was introduced by Troisi et al. who developed a 37-item Ethological Coding System for Interviews (ECSI) [11], based on the work of Grant, McGuire and colleagues, as well as Schelde et al. [6,7,12,13]. Troisi and coworkers observed that unmedicated young males with schizophrenia could be distinguished from normal controls on the basis of their behavioural repertoire during interviews, including the use of "prosocial" behaviours such as yes-nodding and smiling, the use of gestures, and the amount of so-called "displacement activities" as nonverbal signals of motivational conflict [11]. Even though facial and bodily expressivity of patients with schizophrenia may be influenced by neuroleptic dosage [14], or the presence of negative symptoms [15], it has recently been shown that SSD patients treated with second-generation antipsychotics are reduced in their expressive behaviour relative to controls, even when medication is taken into account [16]. In particular, patients with SSD send fewer signals of affiliation with others, fewer affirmative expressions, and fewer signals that convey the meaning of appeasement, which serve the purpose to reduce interpersonal aggression. In general, such prosocial patterns of behaviour are seen as nonverbal signals that invite social interaction, which at least some patients with SSD lack or use to a very limited extent [3,16]. This reduction of non-verbal expressivity, particularly the lack of prosocial signals, predicts functional disability in patients with schizophrenia [17], and correlates to a certain extent with standard psychopathology measures, simply because such nonverbal behaviour constitutes an important aspect of clinicians' ratings of symptom severity [16].

Poor social competence in social interaction, in turn, has been found to be uniquely associated with impaired social cognition in patients with schizophrenia. Specifically, Brüne (2005) and Brüne et al. (2007) demonstrated in independent samples of patients with schizophrenia and SSD that an impaired ability to appreciate the mental states of others, commonly referred to as "mentalising" or "theory of mind", predicted poor social skills in patients better than non-social cognition such as executive functioning or intelligence [18,19]. Previous research has revealed that patients with SSD are highly compromised in their ability to comprehend mental states of others [20]. Impaired mentalising abilities are most prominent in patients with negative symptoms or disorganised behaviour and partly independent of cognitive functioning [21-24]. Moreover, a mentalising deficit also pertains to the broader schizophrenia phenotype and has been demonstrated in otherwise healthy subjects with high schizotypy scores [25,26].

With regards to the association of non-verbal expressivity with social functioning on one hand, and the link of social cognition with social competence in SSD on the other hand, it is conceivable that patients' reduced prosocial expressivity is associated with their poor understanding of others' minds. For example, a patient who is unable to take into account her interlocutor's knowledge, intentions, or desires that are different from her own may perhaps (unconsciously) withhold nonverbal signals that are usually expressed to facilitate communication and to underscore the meaning of what is transmitted verbally. The idea that reduced nonverbal expressivity could be linked with patients' social-cognitive impairments and their poor social competence has, to the best of our knowledge, not been empirically examined so far. It is, of course, unlikely that complex interpersonal behaviours can be completely reduced to a single factor such as the ability to attribute mental states to others. In any event, we were interested in the question whether nonverbal behaviour would at least correlate with independent raters' impression of patients' social skills, standard measures of psychopathology, and particularly social (as well as non-social) neurocognition. Specifically, we hypothesised that patients with SSD would differ from healthy controls in terms of non-verbal expressivity and (social and non-social) neurocognition; we further hypothesised that the SSD patients with poor prosocial behaviour would display reduced social skills in social interaction, as independently rated by nursing staff, and impaired social cognitive abilities relative to patients with preserved nonverbal expressivity, but would not necessarily differ with respect to other (non-social) neurocognitive domains.

## Methods

### Participants

50 in-patients (22 males, 28 females) diagnosed with schizophrenia (N = 38), schizoaffective disorder (N = 9) and delusional disorder (N = 3) according to DSM-IV-TR criteria [27] were included. All patients were in sub-acute stages of their illnesses such that they were able to give full informed consent in writing and to complete the neuropsychological test battery. Patients with a history of substance abuse, severe brain injury or mental retardation were excluded. All patients received second-generation antipsychotic substances (SGA). The mean chlorpromazine equivalent dosage (CPZ) as determined according to Wood's suggestions [28] was 667.73 (SD ± 603 mg) per day. For comparisons, 30 healthy controls (10 males, 20 females) were included, paralleled for age and sex distribution. The study was approved by the Ethics Committee of the University of Bochum.

Patients' mean age was 39.24 (SD ± 13.55), their mean age at onset of SSD was 29.29 years (SD ± 13.99) and their average duration of illness was 9.85 years (SD ± 8.79). The mean age of the control group was 36.83 (SD ± 13.53). No differences between the groups were found with respect to sex distribution (chi^2 ^= .889, df = 1, Fisher's exact test, p = .48, n.s.) or age (t = .77, df = 78, p = .444, n.s.). Group comparisons for demographic variables, cognitive performance as well as PANSS ratings and social competence scores for patients are shown in Table 1.

**Table 1:** Demographic characteristics and performance of patients with SSD and controls

	**Patients**		**Healthy Controls**		**Sign.**
	mean	SD	mean	SD	

***N***	50		30		
**M:F**	22:28		10:20		.480
**Age**	39.24	13.55	36.83	13.53	.444
**MWT (IQ)**	103.9	13.34	111	13.55	.024
**Picture Completion**	11.1	3.48	13.67	2.2	< .001
**Zoo Map**	0	3.23	4.41	3.55	< .001
**WCST pers.**	3.18	3.3	0.93	1.39	< .001
					
**Mechanical**	21.24	2.85	23.27	1.51	.001
**Social script**	21.56	3.07	23.03	2.17	.008
**Capture**	13.82	5.07	16.7	4.17	.014
**False belief**	16.2	5.45	21.43	2.81	< .001
**Sequencing (Brüne)**	28.0	6.65	33.83	2.59	< .001
**Questionnaire**	18.86	3.81	21.9	1.37	< .001
**Mentalising II total**	46.82	8.97	55.53	3.53	< .001
					
**Duration of illness**	9.85	8.79			
**PANSS positive**	14.92	5.49			
**PANSS negative**	17.48	8.62			
**PANSS disorganisation**	22.02	6.36			
**PANSS excitement**	6.42	2.62			
**PANSS affective sympt.**	10.82	3.46			
**PANSS sum**	71.74	16.43			
					
**Social Behaviour**	13.7	8.84			

### Behavioural assessment

#### Ethological assessment of non-verbal expressivity

Non-verbal expressivity of patients and controls was evaluated using the Ethological Coding System for Interviews (ECSI) [3]. The ECSI comprises 37 different patterns of behaviour, eight of which are summarised under the term "prosocial behaviour". Prosocial behaviours embrace both patterns of behaviour that invite and positively reassure social interaction (referred to as "affiliation") and behaviours signalling appeasement that are used to prevent aggression in social interactions (termed "submission"). Accordingly, non-verbal expressions of prosociality included: "head to side" movements; "bob", a sharp upwards movement of the head, similar to an inverted nod; "flash", a quick raising and lowering of the eyebrows; "raise", a movement where the eyebrows are raised and kept up for some time; "smile", where the lip corners are typically drawn back and up; "nod", as affirmative gesture; "lips in", characterised by drawing the lips slightly inwards and pressing the lips together; and "mouth corners back", which describes the drawing back of the corners of the mouth without raising the mouth angles as in smiling, thus, signalling attenuated fear. The ECSI was specifically designed for measuring nonverbal behaviour during interviews based on published human ethograms that have revealed universal non-verbal expressions in social interaction [8,29].

The interviews carried out by three female psychologists were videotaped with a camera such that the subjects' faces were in full view. To ascertain optimal evaluation, two trained observers simultaneously examined the videotapes for the presence or absence of each of the behavioural items in successive 15-second intervals. As suggested by Troisi [3], we used a one-zero (i.e. present-versus-absent in a 15-second interval) sampling method for recording the results, which has been shown to highly correlate with both frequency and duration measures of the same behaviour in previous studies. To avoid distraction of the evaluation process by verbal material the video player was turned mute during the scoring procedure. Moreover, to maximise accuracy, if disagreement between the two raters occurred with regards to any one rating interval, the respective time interval was re-examined until a consensus decision could be achieved. The overall duration of the videotaped part of the interview was 10 minutes (thus, 40 15-second sampling intervals altogether) during which the interviewer collected as much information as possible for rating the subjects' psychopathology using the Positive and Negative Symptom Scale (PANSS) [30]. We chose this setting for both groups to improve comparability of group results, even though this procedure might arguably have created greater or even less emotional involvement in patients compared to healthy controls. For further analyses, the scores of individual behaviours for each subject are expressed as the proportion of intervals during which the behavioural pattern occurred.

#### Social behaviour and social competence

Patients' social behaviour and social competence were rated by an experienced nursing staff member who was most familiar with the patients' actual behaviour in social interactions using the Social Behaviour Scale (SBS) [31]. The SBS represents a 21-item rating scale comprising communicative skills, socially inappropriate behaviours, autistic symptoms (muttering, laughing to self), affective symptoms (anxiety, restlessness, depression), and movement disorders (bizarre behaviour, mannerisms, posturing). Each item is rated according to the severity of deviation on a Likert-type scale ranging from "0" (absent) to "4" (severe).

#### Psychopathology

Psychopathology was rated using the Positive and Negative Syndrome Scale (PANSS) [30]. Here, we chose a novel five-factor model of the PANSS [32] instead of the classic three-factor model, because the former has been shown to have superior statistical validity, and because we were specifically interested in the question whether ratings of nonverbal behaviour and social competence would correlate with any one of these more specific factors (e.g., positive, negative, disorganised, excitement and affective). All ratings of psychopathology and social behaviour were carried out blind to the patients' performance on the social and non-social neurocognitive tasks.

### Neurocognition

#### Non-social cognition

Verbal intelligence was assessed using the German "Mehrfachwahl-Wortschatz-Test", that is, "Multiple Choice Vocabulary Test" (MWT) [33], which resembles the "Spot-the-Word-Test" [34]. The MWT is believed to index premorbid intelligence in patients with psychiatric disorders. Non-verbal intelligence was assessed using the Picture Completion Task, a subtest of the "Wechsler Adult Intelligence Scale", revised German version (WAIS-R) [35].

To assess executive functioning skills we used the Zoo Map Test from the Behavioral Assessment of the Dysexecutive Syndrome battery (BADS) [36] to assess executive planning. The first part of the Zoo Map test requires participants to mentally plan a route through a zoo drawn on a map while taking into account given rules such as not to take a certain trail twice. The second part of the test simply requires participants to follow detailed instructions concerning how to find their way through the zoo terrain. We used the score from the more challenging first part of the Zoo Map Test for further analyses.

Cognitive flexibility was also assessed using a simplified computer version of the Wisconsin Card Sorting Test (WCST) [37]. The number of perseverative errors on this task was used for further analyses.

#### Social cognition

The ability to appreciate mental states was examined first using a non-verbal "false-belief" picture-sequencing task in which, for each false-belief story sequence, four cartoon pictures depict a story character who acts on the basis of a mistaken belief concerning the true location of a certain critical item (e.g. a story character is ignorant about the true location of an item which had been moved or erroneously blames another character for having moved the item). These false-belief sequences were first developed by Langdon et al. [21] and have since been used in non-clinical and clinical schizotypal samples to demonstrate mentalising deficits [25,26]. In addition to the four false-belief sequences, 12 additional sequences, also comprising four pictures per sequence and developed by Langdon and Coltheart [25], depicted "mechanical", "social-script", and "capture" stories. The mechanical sequences illustrated simple physical cause-and-effect events (such as a stone rolling down a slope); the social script sequences depicted interacting characters without the necessity to infer the mental states of these characters; the capture sequences were designed to test the ability to suppress salient misleading information in favour of less salient but more relevant information that eventually led to correct sequencing. In contrast to previous studies using this task, the administration was computerised such that each sequence of four pictures was presented in a jumbled order to the participants on a computer screen. The participants were asked to move the pictures using a computer mouse until they were certain that the sequencing of the four pictures showed a logical order of events. Prior to the experimental sequences, which were presented in a random order for each participant, two practice sequences were presented to ensure that all participants had fully understood the procedure. Scoring was according to Langdon et al.'s suggestions [21], that is, for each sequence, two points were given for the first and last correctly positioned pictures, and one point each for correct positioning of the two middle pictures. Accordingly, participants could obtain a maximum of 6 points per sequence, thus 24 points per sequence type (i.e. false-belief, mechanical, social-script or capture).

In addition, a second cartoon series tapping into mentalising abilities was given to the participants. Six cartoon picture stories depict: (1) two scenarios involving co-operation of two characters, (2) two scenarios illustrating deliberate deception of one character by another, and (3) two scenarios showing two characters cooperating at the cost of a third one. The administration procedure was similar to our previous studies [18,19], with the notable difference that pictures and subsequent questions were depicted on a computer screen instead of the previously used paper and pencil version. As above, each picture story consisted of four cards, which were presented in a mixed-up order. The participants' positioning of pictures was scored as per Langdon et al.'s series. In addition to the non-verbal component of this task (i.e. the sequencing of the pictures), the participants also answered 23 questions probing understanding of the mental states of the story characters. These questions included mentalising questions ranging from first to third-order complexity and requiring true and false correct answers, as well as questions probing the understanding of intended deception, cheating, and cooperation. Whenever the participant failed to sequence the story correctly, the picture story was re-arranged correctly by the experimenter before the questions pertaining to the story were asked. Total scores for sequencing (36 pts. maximum) and for responses to the questionnaire (23 pts. maximum) were calculated (thus 59 pts. altogether).

### Statistical analysis

Wherever skewness and kurtosis of the variables were within acceptable ranges, we used student's t-tests for group comparisons. For non-normally distributed variables we used non-parametric Mann-Whitney-U tests. Univariate analyses of variance with covariates were carried out to examine the specificity of mentalising deficits in the patient group. To examine associations of psychopathology and medication with non-verbal behaviours in the patient group we calculated parametric correlation coefficients. Analyses were performed using SPSS for Macintosh, version 13.0.

## Results

### Between-group differences

Comparisons between patients with SSD and controls revealed significant differences with regards to non-verbal expressivity and neurocognition (both social and non-social). Social competence and psychopathology were rated in patients only. Patients displayed significantly fewer prosocial behaviours (t = -5.072, df = 78, p < .001) – that is, such behaviours were present in only 21.38 percent of the sampling intervals for patients compared to 29.46 percent for controls.

Moreover, in terms of cognitive performance patients with SSD had lower IQ scores, both verbal (premorbidly) (t = -2.308, df = 78, p = .024) and non-verbal (Mann-Whitney-U = 384.5, Z = -3.466, p = .001). They also performed more poorly than controls on the executive planning task (i.e. Zoo Map; t = 5.642, df = 77, p < .001), and were cognitively less flexible (Mann-Whitney-U = 296.0, Z = -4.373, p < .001) compared to controls as determined using the number of perseverative errors in the WCST.

Similar to previous studies, patients with SSD performed more poorly on all sequencing tests of mentalising. On the first sequencing task, patients obtained lower scores on the non-social control stories – mechanical (Mann-Whitney-U = 435.5, Z = -3407, p = .001), social script (Mann-Whitney-U = 514.5, Z = -2.646, p = .008), and capture (Mann-Whitney-U = 503.5, Z = -2.464, p = .014) – as well as on the false belief stories (Mann-Whitney-U = 321.5, Z = -4.292, p < .001). Similarly, patients scored lower on the sequencing part of the Brüne [18,19] cartoons' task (Mann-Whitney-U = 333.5, Z = -4.213, p < .001). They also made more errors in answering the mentalising questions (Mann-Whitney-U = 326.5, Z = -4.276, p < .001), and thus had lower total scores for this task (Mann-Whitney-U = 263.0, Z = -4.855, p < .001).

Although not optimally suitable for non-normally distributed variables, we also conducted ANCOVAs (which are nevertheless very robust procedures) to investigate whether or not the mentalising deficits in the SSD patients were selective, i.e. independent of the non-social cognitive impairment. We therefore entered both measures of IQ, executive functioning variables, and the different non-social sequencing scores (i.e. mechanical, social script and capture scores) as covariates in the equations (one at a time) for both the false-belief picture-sequencing scores and the mentalising score according to Brüne (2005). Notably, the mentalising deficits in SSD remained significantly different from controls in every condition. Table 2 shows the results for each covariate.

**Table 2:** Results of the ANCOVAs concerning selectivity of mentalising deficits in patients compared with controls (upper row: False belief task, lower row: mentalising II task)

**Covariate**	**df**	**Mean square**	**F**	**Statistics**
no covariate	1	513.521	23.794	p < .001
	1	1423.541	25.793	p < .001
MWT IQ	1	389.528	18.647	p < .001
	1	1060.207	20.099	p < .001
Picture completion	1	193.606	10.599	p = .002
	1	467.812	10.899	p = .001
WCST pers.	1	219.384	12.430	p = .001
	1	550.198	12.262	p = .001
Zoo Map	1	129.732	6.565	p = .012
	1	382.938	7.671	p = .007
Mechanical	1	208.275	11.615	p = .001
		697.903	14.168	p < .001
Social script	1	358.326	17.886	p < .001
	1	947.784	19.246	p < .001
Capture	1	265.561	15.493	p < .001
	1	928.654	18.313	p < .001

MWT: Mehrfachwahl-Wortschatz-Test (verbal or premorbid IQ); WCST pers.: number of perseverative errors in the Wisconsin Card Sorting Test.

### Correlations within the patient group

To determine associations of non-verbal expressivity with measures of social competence, neurocognitive variables (social and non-social) and standard psychopathological ratings within the patient group, we calculated parametric correlation coefficients. Accordingly, non-verbal expressivity correlated negatively with the PANSS disorganisation score (r = -.294, p = .038). There were also negative associations of prosocial behaviour with the PANSS positive symptoms score (r = -.256, p = .073), with the PANSS negative symptom score (r = -.273, p = .055), with Langdon's false-belief sequencing score (r = .241, p = .091), with the questionnaire part of Brüne's mentalising task (r = .26, p = .069), with the total mentalising score from Brüne's task (r = .236, p = .099), and with the social behaviour score as determined using the SBS (r = -.273, p = 0.69); however, all these associations failed to reach statistical significance. By contrast, there were multiple correlations between measures from the two sequencing tasks, between neurocognitive measures and the social behaviour score, and, in part, with psychopathology. Specifically, only Langdon's false-belief sequencing score (r = -.479, p = .001) and Brüne's mentalising scores (all p's < .01) correlated significantly with the social behaviour score, as did the number of perseverative errors on the WCST (r = -.524, p < .001). Moreover, the PANSS disorganisation score correlated significantly with several of the sequencing task scores and with the social behaviour measure. Medication levels did not correlate with any one of the other variables (correlations are displayed in additional file 1).

### Differences between patients with low and high levels of non-verbal expressivity

Since we were particularly interested in the possible associations between nonverbal expressivity and both social competence and social cognitive performance of patients with SSD, and in light of the relatively weak correlations of non-verbal expressivity with these various measures, we compared the patients falling into the lowest quartile with the patients falling into the highest quartile of non-verbal expressivity with regards to the neurocognitive (social and non-social) and behavioural measures. We chose this procedure based on the hypothesis that patients who scored lowest according to the ethological coding system would perhaps do so because of more difficulties in understanding other minds, when compared to patients with high scores on non-verbal expressivity, who, according to our hypothesis would have a preserved understanding of other minds, and would therefore be relatively unimpaired in social interaction.

Accordingly, patients with the least prosocial behaviour (N = 14) displayed, on average, prosocial expressions of affiliation or submission in only 11.9 per cent (± 1.8) of observed intervals. In contrast, patients with high levels of prosocial behaviour (N = 12) showed affiliative or submissive expressions in 33.41 per cent (± 4.04) of intervals. Thus, high "prosocials" were well within the range of nonverbal expressivity of healthy controls.

Most interestingly, when comparing low prosocials with high prosocials within the patient sample, we found a significant group difference with regards to the false-belief sequencing score (t = -2.041, df = 24, p = .05), while the group difference for the total sequencing and questionnaire score from the second mentalising task approached significance (t = -2.007, df = 24, p = .06). In contrast, no differences between high and low prosocial patients emerged in any one of the other cognitive tasks. As would be expected, however, the patient groups differed with regards to psychopathology scores (i.e. PANSS positive, negative, and disorganised subscores). In addition, low prosocial patients had significantly less social competence, that is, higher scores on the SBS, compared with patients with high prosocial expressivity (t = 2.133, df = 20, p = .05). Results are summarised in Table 3.

**Table 3:** Comparison of task performance between patients with low non-verbal expressivity ("low prosocials") and patients with high (normal) nonverbal expressivity ("high prosocials")

**Parameter**		**Group**	**Mean**	**SD**	**sign.**
**Age**		Low prosocials	41.29	14.66	p = .131
		High prosocials	33.5	9.78	
**Duration of illness**		Low prosocials	9.93	9.4	p = .731
		High prosocials	8.73	7.36	
**Intelligence**	MWT	Low prosocials	102.64	11.93	p = .502
		High prosocials	106.25	15.06	
	Picture completion	Low prosocials	11.07	3.08	p = .993
		High prosocials	11.08	4.23	
**Executive functioning**	WCST pers.	Low prosocials	3.69	2.84	p = .109
		High prosocials	2.17	1.47	
	Zoo Map	Low prosocials	-0.93	3.95	p = .075
		High prosocials	1.75	3.28	
**Langdon et al.'s sequencing tasks**	Mechanical	Low prosocials	21.07	2.9	p = .087
		High prosocials	22.67	1.5	
	Social script	Low prosocials	20.64	2.85	p = .075
		High prosocials	22.75	2.93	
	Capture	Low prosocials	13.5	4.78	p = .419
		High prosocials	15.08	5.02	
	False belief	Low prosocials	15.86	4.88	p = .05
		High prosocials	19.75	4.81	
**Mentalising Task II**	Sequencing	Low prosocials	27.5	6.67	p = .133
		High prosocials	31.08	4.72	
	Questionnaire	Low prosocials	18.43	3.84	p = .071
		High prosocials	20.83	2.33	
	Total score	Low prosocials	45.57	9.04	p = .06
		High prosocials	51.83	6.38	
**Positive and Negative Symptom Scale**	positive	Low prosocials	18.43	4.59	p = .004
		High prosocials	13.17	3.74	
	negative	Low prosocials	21.07	7.77	p = .027
		High prosocials	14.5	6.23	
	disorganised	Low prosocials	24.07	5.66	p = .007
		High prosocials	17.83	4.97	
	excitement	Low prosocials	5.86	1.66	p = .546
		High prosocials	6.25	1.6	
	affective	Low prosocials	12.07	3.79	p = .111
		High prosocials	9.82	2.27	
**Social Competence (SBS)**		Low prosocials	16.64	10.41	p = .05
		High prosocials	8.73	6.54	
**Chlorpromazine equivalents**		Low prosocials	794.14	838.91	p = .551
		High prosocials	621.09	491.64	

MWT: Mehrfachwahl-Wortschatz-Test (verbal or premorbid IQ); WCST pers.: number of perseverative errors in the Wisconsin Card Sorting Test.

## Discussion

In the present study we sought to explore the associations of nonverbal expressive behaviour with social competence and neurocognition (social and non-social) in patients with SSD. Specifically, we were interested in the question whether reduced non-verbal expression of behaviours that invite social communication, referred to as prosocial behaviours [3], would be linked with poorer social competence and impaired understanding of mental states in patients with SSD.

In line with previous studies [11,16], we were able to show that patients with SSD were reduced in their non-verbal expressivity during an interview that aimed at exploring psychopathological symptoms and subjective factors of distress when compared to controls, paralleled for age and gender, who were observed during a similar interview. Moreover, patients' social competence was associated with levels of executive functioning and mentalising abilities, a finding that mirrors previous results from a clinical sample that overlaps somewhat with the present study [19]. As expected, patients were also significantly less able than controls to appreciate the mental states of story characters. The mentalising deficit appeared to be selective in SSD, although the statistical analysis of covariance was not optimally suited for the evaluation of this question. Nevertheless the follow-up comparison of low and high prosocial patients was consistent with the suggestion of selectivity of mentalising deficit in the patients.

The most important question of the present study was, however, to examine whether or not patients' nonverbal expressive behaviour would be associated with their social competence as rated by carers who were oblivious to the study design, and whether nonverbal expressivity would be linked with the patients' neurocognitive (social and non-social) abilities. The basic hypotheses motivating this question were that patients with SSD who are less able to understand others' minds would display not only more behavioural abnormalities as measured using the Social Behaviour Scale [31], but also reduced nonverbal expressivity that normally invites social interaction, i.e. prosocial behaviours. Both hypotheses were partially confirmed. Even though nonverbal expressivity did not correlate with any one of the neurocognitive (social or non-social) variables or with social competence at a 0.05 significance level, we found that patients with the lowest level of nonverbal expressivity performed more poorly on a false-belief mentalising task compared with patients whose nonverbal expressivity during interviews was within the range of healthy controls. Moreover, the association of reduced prosociality with scores from a second mentalising task approached significance. In contrast, all other differences between low and high prosocials were inconspicuous (perhaps with the exception of executive planning, where the level of significance was .075). As expected, there were also significant differences between low and high prosocials with regards to standard measures of psychopathology, which underscores the relevance of patients' nonverbal behaviour for clinicians' ratings of symptom severity [16]. Moreover, low and high prosocials also differed significantly in social behavioural skills. These associations are entirely compatible with the finding reported elsewhere that impaired mentalising is the best predictor of poor social competence in patients with SSD and has greater explanatory power than executive functioning or IQ [19]. Notably, in the present study no correlation of any one variable was found with medication levels, which can perhaps best be accounted for by treatment with second-generation antipsychotic substances.

To the best of our knowledge, this is the first study to link nonverbal expressive behaviour in patients with SSD with other measures of interpersonal functioning and neurocognitive (social and non-social) performance. The finding that nonverbal expressivity could be associated with the ability to mentalise has not been previously reported. There has been considerable speculation about the cause of reduced behavioural expressivity in patients with SSD. The most obvious though superficial explanation is that reduced expressivity is a direct consequence of the severity of negative symptoms, a notion that is partially consistent with our results [38]. However, the largest negative correlation with regards to standard psychopathology measures was found between nonverbal expressivity and patients' level of behavioural and cognitive disorganisation and not with the level of negative symptoms. Previous studies [24,39] and a recent meta-analysis [23] have revealed that mentalising is most severely impaired in patients with disorganised symptomatology. It is therefore conceivable that poor mentalising abilities contribute to patients' reduced use of nonverbal behaviours that invite and sustain social interaction in dyadic conversations, and that reduced signals of this type then contribute, in part, to clinicians' severity ratings of negative symptoms during (dyadic) clinical interviews.

## Limitations

The present study does not allow causal explanations. Rather, it is limited in explanatory power to correlational associations and differences between extremes of variation with regards to nonverbal expressivity within the patient group. This might in the first place be a result of the heterogeneity of the sample in terms of symptomatology. Moreover, findings may not be generalisable to patients in symptomatic remission. Ideally (and this ought to be addressed in future studies), a similar study in a more homogenous sample of patients with prominent disorganised symptoms may yield even more promising results. Likewise, a future study that allows for finer-grained discrimination of severities of different types of negative symptoms would be informative. Finally, it would be interesting to explore in greater detail how non-verbal expressivity is related to self-reflection, because self-reflexive awareness has been shown to play a role in both attribution of mental states to others and social functioning in SSD and other severe mental disorders [40].

## Conclusion

Linking neurocognition (particularly social cognition) with real-world behaviour (particularly nonverbal expression) has been a relatively neglected issue in clinical research to date. The present study is thus important in seeking to better specify the factors that actually guide and motivate patients' interpersonal nonverbal behaviour.

## List of abbreviations

ANCOVA: Analysis of Covariance; BADS: Behavioral Assessment of the Dysexecutive Syndrome; CPZ: Chlorpromazine equivalent dosage; DSM-IV-TR: Diagnostic and Statistical Manual, Fourth Edition, Text Revision; ECSI: Ethological Coding System for Interviews; EM-FACS: Emotional Facial Action Coding System; IQ: Intelligence quotient; MWT: Mehrfachwahl-Wortschatztest; PANSS: Positive and Negative Syndrome Scale; SBS: Social Behaviour Scale; SD: Standard deviation; SGA: Second generation antipsychotics; SPSS: Statistical Package for the Social Sciences; SSD: schizophrenia spectrum disorders; WAIS-R: Wechsler Adult Intelligence Scale – Revised; WCST: Wisconsin Card Sorting Test.

## Competing interests

The authors declare that they have no competing interests.

## Authors' contributions

MB conceived and designed the study and wrote the manuscript. MA-H, CS and CL assessed patients and controls, evaluated the participants' nonverbal behaviour and contributed to the statistical analyses. RL wrote the manuscript and designed one of the mentalising tasks used in the study.

## Supplementary Material

Additional file 1 **Correlations of behavioural and demographic variables within the patients group**. Correlation coefficients of neurocognitive and behavioural measures within the patient group are displayed in a separate table. Click here for file

## References

[B1] Pitman RK, Kolb B, Orr SP, Singh MM (1987). Ethological study of facial behavior in non paranoid and paranoid schizophrenic patients. Am J Psychiatry.

[B2] Krause R, Steimer E, Sänger-Alt C, Wagner G (1989). Facial expression of schizophrenic patients and their interaction partners. Psychiatry.

[B3] Troisi A (1999). Ethological research in clinical psychiatry: the study of non verbal behavior during interviews. Neurosci Biobehav Rev.

[B4] Friesen WV, Ekman P (1978). Facial Action Coding System.

[B5] Gaebel W, Wölwer W (2004). Facial expressivity in the course of schizophrenia and depression. Eur Arch Psychiat Clin Neurosci.

[B6] McGuire MT, Polsky RH (1979). Behavioral changes in hospitalized acute schizophrenics. An ethological perspective. J Nerv Ment Dis.

[B7] McGuire MT, Polsky RH (1983). Sociospatial behavioral relationships among hospitalized psychiatric patients. Psychiat Res.

[B8] Eibl-Eibesfeldt I (1995). Die Biologie menschlichen Verhaltens Grundriss der Humanethologie Piper.

[B9] Manstead ASR, Fischer AH, Jakobs EB, Philippot P, Feldman RS, Coats EJ (1999). The social and emotional functions of facial displays. The Social Context of Nonverbal Behavior.

[B10] Schmidt KL, Cohn JF (2001). Human facial expressions as adaptations: evolutionary questions in facial expressions research. Yearb Phys Anthropol.

[B11] Troisi A, Spalletta G, Pasini A (1998). Non-verbal behaviour deficits in schizophrenia: An ethological study of drug-free patients. Acta Psychiat Scand.

[B12] Grant EC (1968). An ethological description of non-verbal behaviour during interviews. Br J Med Psychol.

[B13] Schelde JTM, Pedersen J, Hannibal E, Behnke K, Nielsen BM, Hertz M (1988). An ethological analysis of depression: Comparison between ethological recording and Hamilton's rating of five endogenously depressed patients. Acta Psychiat Scand.

[B14] Schneider F, Ellgring H, Friedrich J, Fus I, Beyer T, Heimann H, Himer W (1992). The effects of neuroleptics on facial action in schizophrenic patients. Pharmacopsychiat.

[B15] Trémeau F, Malaspina D, Duval F, Correa H, Hager-Budny M, Coin-Bariou L, Macher JP, Gorman JM (2005). Facial expressiveness in patients with schizophrenia compared to depressed patients and nonpatient comparison subjects. Am J Psychiatry.

[B16] Brüne M, Sonntag C, Abdel-Hamid M, Lehmkämper C, Juckel G, Troisi A (2008). Non-verbal behavior during standardized interviews in patients with schizophrenia spectrum disorders. J Nerv Ment Dis.

[B17] Troisi A, Pompili E, Binello L, Sterpone A (2007). Facial expressivity during the clinical interview as a predictor of functional disability in schizophrenia. A pilot study. Progr Neuropsychopharmacol Biol Psychiat.

[B18] Brüne M (2005). Emotion recognition, 'theory of mind' and social behavior in schizophrenia. Psychiat Res.

[B19] Brüne M, Abdel-Hamid M, Lehmkämper C, Sonntag C (2007). Mental state attribution, neurocognitive functioning, and psychopathology: What predicts poor social competence in schizophrenia best?. Schizophr Res.

[B20] Harrington L, Siegert R, McClure J (2005). Theory of mind in schizophrenia: a critical review. Cogn Neuropsychiat.

[B21] Langdon R, Michie PT, Ward PB, McConaghy N, Catts S, Coltheart M (1997). Defective self and/or other mentalising in schizophrenia: a cognitive neuropsychological approach. Cogn Neuropsychiat.

[B22] Langdon R, Coltheart M, Ward PB, Catts SV (2001). Mentalising, executive planning and disengagement in schizophrenia. Cogn Neuropsychiat.

[B23] Sprong M, Schothorst P, Vos E, Hox J, van Engeland E (2007). Theory of mind in schizophrenia. Br J Psychiatry.

[B24] Abdel-Hamid M, Lehmkämper C, Sonntag C, Juckel G, Daum I, Brüne M Theory of mind in schizophrenia: The role of clinical symptomatology and neurocognition in understanding other people's thoughts and intentions. Psychiat Res.

[B25] Langdon R, Coltheart M (1999). Mentalising, schizotypy, and schizophrenia. Cognition.

[B26] Langdon R, Coltheart M (2004). Recognition of metaphor and irony in young adults: the impact of schizotypal personality traits. Psychiat Res.

[B27] American Psychiatric Association (2000). Diagnostic and Statistical Manual of Mental Disorders, text revision.

[B28] Woods SW (2003). Chlorpromazine equivalent doses for newer atypical antipsychotics. J Clin Psychiat.

[B29] Brannigan CR, Humphries DA, Blurton Jones N (1974). Human non-verbal behaviour, a means of communication. Ethological studies of child behaviour.

[B30] Kay SR, Opler LA, Lindenmayer JP (1989). The Positive and Negative Syndrome Scale (PANSS): rationale and standardisation. Br J Psychiatry.

[B31] Wykes T, Sturt E (1986). The measurement of social behaviour in psychiatric patients: an assessment of the reliability and validity of the SBS schedule. Br J Psychiatry.

[B32] Gaag M Van der, Hoffman T, Remijsen M, Hijman R, de Haan L, van Meijel B, van Harten PN, Valmaggia L, de Hert M, Cuijpers A, Wiersma D (2006). The five-factor model of the Positive and Negative Syndrome Scale II: A ten-fold cross-validation of a revised model. Schizophr Res.

[B33] Lehrl S, Triebig G, Fischer B (1995). Multiple choice vocabulary test MWT as a valid and short test to estimate premorbid intelligence. Acta Neurol Scand.

[B34] Baddeley A, Emslie H, Nimmo-Smith I (1993). The Spot-The-Word Test: a robust estimate of verbal intelligence on lexical decision. Br J Clin Psychol.

[B35] Tewes U (1991). Hamburg-Wechsler-Intelligenztest für Erwachsene – Revision 1991.

[B36] Wilson BA, Alderman N, Burgess P, Emslie H, Evans JJ (1996). Behavioural Assessment of the Dysexecutive Syndrome (BADS).

[B37] Nelson HE (1976). A modified card sorting test sensitive to frontal lobe defects. Cortex.

[B38] Troisi A, Pasini A, Bersani G, Di Mauro M, Ciani N (1991). Negative symptoms and visual behavior in DSM-III-R prognostic subtypes of schizophreniform disorder. Acta Psychiat Scand.

[B39] Sarfati Y, Hardy-Baylé MC, Brunet E, Widlöcher D (1999). Investigating theory of mind in schizophrenia: influence of verbalization in disorganized and non-disorganized patients. Schizophr Res.

[B40] Dimaggio G, Lysaker P, Carcione A, Nicolò G, Semerari A (2008). Know yourself and you shall know the other ... to a certain extent: multiple paths of influence of self-reflection on mindreading. Consc Cogn.

