# Supplementary material for: Linking social cognition with social interaction: Non-verbal expressivity, social competence and "mentalising" in patients with schizophrenia spectrum disorders
Source: Behav Brain Funct. 2009 Jan 23;5:6. doi: 10.1186/1744-9081-5-6 (PMC2637294; doi:10.1186/1744-9081-5-6)
Supplement: Additional file 1 — Correlations of behavioural and demographic variables within the patients group. Correlation coefficients of neurocognitive and behavioural measures within the patient group are displayed in a separate table. [file 1744-9081-5-6-S1.doc]

**Additional file:** Correlations of behavioural and demographic variables within the patients group

|  | Age | DOI | MWT (IQ) | PC (IQ) | WCST  pers. | Zoo Map | Non-verbal | SBS | PANSS pos. | PANSS neg. | PANSS dis. | PANSS exc. | PANSS affect. | Mechanical | Social Script | Capture | FB | Ment. II Sequ. | Ment. II Quest. | Ment. II Sum | CPZ |
| --- | --- | --- | --- | --- | --- | --- | --- | --- | --- | --- | --- | --- | --- | --- | --- | --- | --- | --- | --- | --- | --- |
| Age |  | .238  .104 | .168  .244 | -.093  .530 | .230  .111 | -.112  .437 | -.170  .238 | -.044  .773 | .182  .205 | -.244  .087 | .005  .973 | -.060  .677 | -.270  .058 | -.312  .027 | -.123  .394 | -.001  .997 | -.280  .049 | -.356  .011 | -.142  .325 | -.327  .020 | .083  .575 |
| DOI |  |  | .109  .459 | .031  .837 | .116  .437 | .037  .800 | .007  .963 | .295  .055 | .102  .489 | .195  .185 | .178  .226 | .039  .793 | .034  .817 | -.123  .406 | -.103  .486 | -.108  .465 | -.263  .070 | .129  .381 | -.040  .787 | .087  .556 | -.104  .490 |
| MWT  (IQ) |  |  |  | .463  .001 | -.271  .060 | .309  .029 | .207  .149 | -.257  .089 | -.088  .544 | -.266  .062 | -.080  .581 | .169  .240 | -.156  .280 | .075  .606 | .032  .825 | .493  <.001 | .254  .075 | .160  .268 | .451  .001 | .307  .030 | -.210  .152 |
| PC (IQ) |  |  |  |  | -.560  <.001 | .367  .010 | .020  .894 | -.229  .139 | .168  .254 | .039  .791 | -.175  .235 | -.211  .150 | -.068  .648 | .240  .101 | .458  .001 | .408  .004 | .427  .002 | .407  .004 | .444  .002 | .489  <.001 | -.027  .860 |
| WCST  pers. |  |  |  |  |  | -.471  .001 | -.167  .252 | .272  .074 | -.064  .664 | .162  .267 | .174  .231 | -.090  .537 | .079  .590 | -.192  .186 | -.141  .336 | -.233  .107 | -.509  <.001 | -.340  .017 | -.661  <.001 | -.503  <.001 | -.009  .952 |
| Zoo Map |  |  |  |  |  |  | .227  .113 | -.524  <.001 | -.107  .460 | -.169  .240 | -.236  .099 | .075  .606 | -.055  .705 | .288  .043 | .404  .004 | .375  .007 | .505  <.001 | .355  .011 | .393  .005 | .442  .001 | .031  .834 |
| Non-verbal expressivity |  |  |  |  |  |  |  | -.273  .069 | -.256  .073 | -.273  .055 | -.294  .038 | .077  .594 | -.172  .231 | .109  .452 | .137  .344 | .171  .235 | .241  .091 | .173  .229 | .260  .069 | .236  .099 | -.105  .476 |
| SBS |  |  |  |  |  |  |  |  | .398  .007 | .438  .003 | .580  <.001 | .174  .254 | .308  .040 | -.138  .366 | -.218  .150 | -.281  .061 | -.479  .001 | -.399  .007 | -.484  .001 | -.511  <.001 | -.033  .831 |
| PANSS  positive |  |  |  |  |  |  |  |  |  | .240  .093 | .385  .006 | .058  .690 | .109  .451 | -.218  .129 | -.091  .531 | -.168  .244 | -.156  .280 | -.142  .327 | -.051  .723 | -.153  .288 | .095  .523 |
| PANSS  negative |  |  |  |  |  |  |  |  |  |  | .239  .095 | -.329  .020 | .376  .007 | -.095  .511 | -.065  .652 | -.325  .021 | -.206  .151 | -.144  .319 | -.259  .069 | -.207  .148 | -.086  .563 |
| PANSS  disorganised |  |  |  |  |  |  |  |  |  |  |  | .389  .005 | .143  .321 | -.246  .084 | -.473  .001 | -.259  .070 | -.361  .010 | -.330  .019 | -.259  .069 | -.373  .008 | -.075  .613 |
| PANSS  excitement |  |  |  |  |  |  |  |  |  |  |  |  | -.028  .849 | -.161  .263 | -.345  .014 | .089  .540 | -.080  .579 | -.125  .386 | -.131  .364 | -.156  .280 | -.067  .649 |
| PANSS affective |  |  |  |  |  |  |  |  |  |  |  |  |  | .127  .381 | -.175  .224 | -.032  .824 | .098  .497 | -.075  .602 | -.042  .771 | -.074  .609 | .121  .414 |
| Mechanical |  |  |  |  |  |  |  |  |  |  |  |  |  |  | .467  .001 | .329  .020 | .420  .002 | .391  .005 | .219  .126 | .384  .006 | -.011  .940 |
| Social script |  |  |  |  |  |  |  |  |  |  |  |  |  |  |  | .239  .094 | .365  .009 | .467  .001 | .068  .639 | .388  .005 | -.060  .686 |
| Capture |  |  |  |  |  |  |  |  |  |  |  |  |  |  |  |  | .445  .001 | .328  .020 | .306  .030 | .369  .008 | .132  .373 |
| False belief |  |  |  |  |  |  |  |  |  |  |  |  |  |  |  |  |  | .426  .002 | .453  .001 | .509  <.001 | -.043  769 |
| Mental. II sequencing |  |  |  |  |  |  |  |  |  |  |  |  |  |  |  |  |  |  | .432  .002 | .928  <.001 | .013  .932 |
| Mental. II questionnaire |  |  |  |  |  |  |  |  |  |  |  |  |  |  |  |  |  |  |  | .727  <.001 | .136  .356 |
| Mental. II sum |  |  |  |  |  |  |  |  |  |  |  |  |  |  |  |  |  |  |  |  | .058  .694 |
| CPZ |  |  |  |  |  |  |  |  |  |  |  |  |  |  |  |  |  |  |  |  |  |

CPZ: Chlorpromazine equivalents; DOI: duration of illness; FB: false belief; IQ: Intelligence quotient; MWT: Mehrfachwahl-Wortschatz-Test; PANSS: Positive and Negative Symptom Scale; PC: Picture completion task; SBS: Social Behaviour Scale; WCST: Wisconsin Card Sorting Test
